# Absorption of bioactive peptides following collagen hydrolysate intake: a randomized, double-blind crossover study in healthy individuals

**DOI:** 10.3389/fnut.2024.1416643

**Published:** 2024-08-01

**Authors:** Nicolina Virgilio, Christiane Schön, Yvonne Mödinger, Bastiaan van der Steen, Sara Vleminckx, Frédérique L. van Holthoon, Anne J. Kleinnijenhuis, Catarina I. F. Silva, Janne Prawitt

**Affiliations:** ^1^Rousselot BV, Ghent, Belgium; ^2^BioTeSys GmbH, Esslingen, Germany; ^3^Triskelion, Utrecht, Netherlands

**Keywords:** bioactive peptides, bioavailability, collagen hydrolysate, free and total hydroxyproline, Pro-Hyp

## Abstract

**Background:**

Collagen hydrolysates (CH) in functional foods and supplements are dietary sources of amino acids (AAs) and di-and tripeptides linked to various health benefits. This study aimed to investigate the single-dose bioavailability of skin- and hide-derived CH from fish, porcine and bovine origin with different molecular weights (bovine 2,000 and 5,000 Da).

**Methods:**

A randomized, double-blind crossover clinical study was performed with healthy volunteers assessing the plasma concentration of free and peptide-bound hydroxyproline (Hyp) as well as selected peptides reported to be abundantly present in collagen.

**Results:**

The pharmacokinetic endpoints demonstrated comparable uptake of free Hyp from all CH. A higher amount of total compared to free Hyp indicated the uptake of substantial amounts of Hyp-containing di- or tripeptides.

**Conclusion:**

Independently of source and molecular weight, all CH yielded relevant plasma concentrations of the investigated metabolites. Larger studies are needed to estimate an ideal level of selected circulating metabolites needed to trigger distinct physiological reactions in target tissues.

## 1 Introduction

Collagen hydrolysates (CH) are produced through extraction and hydrolysis of collagen-rich raw materials such as bone, hide and skin from various animal species. The types of collagen present varies among tissues and the specific collagen sequence is determined by the animal source. Collagen is an evolutionary conserved protein across species, yet as a natural product, differences can be expected between CH from different raw material origins. The manufacturing process produces a complex mixture of peptides of varying lengths and identities. The degree of hydrolysis determines the average peptide length, quantifiable as the mean molecular weight. It is important to note that the molecular weight does not specify peptides’ identity but is determined by the sequence of amino acids (AAs). The oligopeptides generated during the CH manufacturing process are further cleaved during the gastrointestinal passage (1–3). Previously, it was assumed that CH are fully broken down into single AAs during absorption into the blood stream, serving primarily as building blocks for protein synthesis. However, recent studies confirm that both free AAs and bioactive di- and tripeptides appear in the human bloodstream after CH ingestion (4, 5). This is supported by the presence of a specific transport system, PepT1, which contributes to trans-cellular movement of di- and tripeptides across the enterocyte (6). Thus, CH are an important source of bioactive peptides, which, once cleaved from the parent collagen protein and absorbed into the bloodstream, can exert beneficial effects on body functions beyond their nutritional value (1, 7–10). CH-derived bioactive peptides support the strength, structure and elasticity of key extracellular matrix components in cartilage, joints, ligaments and tendons (1, 11–13), bone (14–16), skeletal muscle (17, 18), and skin (19–21). They achieve this by modulating cell functions linked to matrix protein synthesis (22, 23), growth, differentiation and morphogenesis. Additionally, anti-thrombotic (24), anti-hypertensive (23, 25, 26), and neuroprotective (27) functionalities of CH-derived peptides have been described, highlighting their broad potential as functional ingredients for food and dietary supplements.

Collagen is characterized by a distinct AAs composition, unusually high in glycine (Gly) and proline (Pro), and is one of the few animal proteins that contains hydroxyproline (Hyp) (28). The characteristic collagen motif consists of a repeating (Gly-Xaa-Yaa) n sequence, where Xaa and Yaa can be any AAs but are often Pro (28%) and its post-translational modification Hyp (38%) making Gly-Pro-Hyp the most common triplet (10.5%) in the collagen sequence (29). Due to its high abundance, Hyp can be used as a marker to trace collagen in food or blood samples (30). The unique ring structures of Pro and Hyp distinguish them from other AAs in terms of rigidity, chemical stability and biochemical reactions (31), allowing them to form bonds with adjacent AAs that resist hydrolysis by digestive enzymes. This is confirmed by the presence of Hyp-containing di- and tripeptides in the blood after collagen ingestion (4, 32–34). Kleinnijenhuis et al. described changes in size distribution and peptide patterns of CH during digestion and absorption, confirming that Hyp-Gly and especially Pro-Hyp significantly contribute to the total Hyp increase in blood after CH ingestion (5).

Despite the growing knowledge on collagen-derived signature peptides and their individual bioactivities, the identity of all peptides in the complex mix of CH products is not fully described, and the detailed changes these peptides undergo during digestion and absorption are generally unknown. This renders the attempts to elucidate the molecular basis of a CH’s biological activity extremely difficult. Consequently, whether the varying bioactivity of CH products is related to differences in peptide identity and bioavailability, differences in the raw material source, molecular weight (i.e., average peptide length before ingestion), or other elements of the product’s manufacturing process is not well understood. Recent studies have compared the bioavailability of Hyp-containing peptides from various collagen sources, focusing on fish, pork and chicken-derived CH (4, 35, 36). However, bovine-hide derived CH is the predominantly used product in much of the world, particularly the Western hemisphere. To our knowledge, there is no comprehensive study comparing the bioavailability of the most common skin- and hide-derived CH from fish, pork and bovine sources.

To investigate the impact of the raw material source and the molecular weight (2,000 and 5,000 Da, bovine) on the bioavailability, we compared fish skin, porcine skin and bovine hide-derived CH in a single study. This involved targeted analysis of highly abundant and collagen-typical AAs (Hyp) and peptides (Pro-Hyp, Hyp-Gly, Gly-Pro-Hyp, Pro-Gly, Gly-Pro) in human blood after CH ingestion. Additionally, systemically available free and total Hyp were measured to estimate the ratio of uptake in form of free AAs or peptides.

## 2 Materials and methods

### 2.1 Study design

The bioavailability of CH was investigated after single-dose intake using a randomized, double-blind crossover design with a wash-out phase of 7 days between study days. The clinical study was performed at BioTeSys GmbH, Esslingen, Germany. The clinical study was advised and approved by the ethics committee of the Landesärztekammer Baden-Württemberg (F-2019-075) and registered at ClinicalTrials.gov (NCT04097808). It was conducted in accordance with the Declaration of Helsinki and Good Clinical Practice. All volunteers signed an informed consent form prior to the start of the study. The study was performed as pilot study with a sample size of *n* = 6 volunteers. Based on the inclusion criteria, the study collective included healthy non-smoking individuals between 19 and 50 years, with a body mass index (BMI) of ≥19 and ≤ 28 kg/m^2^ and an overall good physical and mental health as established by medical history, physical examination, electrocardiogram, vital signs and results of biochemistry and hematology.

Following an overnight fast of at least 10 h, baseline blood samples were taken. After the baseline sampling, the single-dose study product was ingested according to a randomization scheme. After administration, at 60, 120, 180, 240, and 360 min post, blood samples were collected via an indwelling catheter in the vein in the elbow or forearm. A standardized, low protein meal was served 240 min post dosing (Pretzel + butter). Blood was collected in Monovettes (Sarstedt AG & Co. KG, Nümbrecht, Germany).

EDTA-blood was centrifuged at 3,000 × *g* for 10 min at 4°C. Protein low-bind material was used for preparation of samples and for storage tubes (LoBind^®^, Eppendorf AG, Hamburg, Germany).

### 2.2 Study products

The investigational products were CH in powder form with a protein content of >90%, commercialized as Peptan^®^ and provided by Rousselot BV, Gent, Belgium. The CH products are manufactured by a standardized process of enzymatically hydrolyzed gelatin. CH were of either bovine hide, porcine skin or fish skin origin of low mean molecular weight (2,000 Da) (LMW), and for the bovine CH additionally high mean molecular weight (5,000 Da) (HMW). The products were provided to the volunteers in a randomized fashion as oral single-dose (10 g) dissolved in 200 mL water. All products were very similar in taste, texture, color and odor. Double-blind performance of the study was ensured.

### 2.3 LC–MS analysis

Ultra-performance liquid chromatography tandem mass spectrometry (UPLC-MS/MS, Xevo TQ-S, Waters, Milford, Massachusetts, United States) was applied to analyze samples. The samples were analyzed using three different methods: (1) free AAs, (2) total AAs, and (3) selected collagen peptides (Pro-Hyp, Hyp-Gly, Gly-Pro-Hyp, Pro-Gly, Gly-Pro). Free and total AAs were analyzed on a Xevo TQ-S (Waters, Milford, Massachusetts, United States), the collagen peptides on a QTRAP 6500 (Sciex, Toronto, Canada), both equipped with an Acquity (Waters) UPLC system. The analytical column was an Acquity HSS T3, 100 × 2.1 mm, 1.8 μm (Waters) for all methods. Briefly, for analysis of free AAs an internal standard (IS) mix of AAs was added to the samples and a mild protein precipitation was performed by adding ice-cold acetonitrile (Biosolve, Valkenswaard, The Netherlands) 1:1 v/v. To the supernatant, borate buffer (pH 8.8, prepared with disodium tetraborate, Thermo Fisher Scientific, Hampton, New Hampshire, United States), disodium ethylenediaminetetraacetic acid (Merck, Rahway, New Jersey, United States) and boric acid (Merck, Rahway, New Jersey, United States) and 6-aminoquinolyl-N-hydroxysuccinimidyl carbamate (AQC; Toronto Research Chemicals Inc., North York, Canada) were added. The mixture was incubated at 55°C for 15 min, diluted and analyzed. For analysis of total AAs, IS mix of AAs, milliQ water (>18 MΩ, Advantage A10, Merck Millipore, St. Louis, Missouri, United States) and hydrochloric acid (Merck, Rahway, New Jersey, United States) to an end concentration of 6 M were added to the samples. After overnight incubation at 95°C and evaporation to dryness, borate buffer and AQC were added. The mixture was incubated at 55°C for 15 min, diluted and analyzed. In methods 1 and 2, Hyp was quantified with calibration samples prepared in solvent, using the analyte/IS ratios. Analysis of peptides was performed according to Kleinnijenhuis et al. (37). Dipeptides/tripeptides were purchased from Bachem (Bubendorf, Switzerland). Stable isotope labeled AQC was synthesized by Syncom (Groningen, Netherlands) and used as IS as previously described by Kleinnijenhuis et al. (37). The plasma samples were quantified using calibration samples prepared in pooled human plasma and the other samples using calibration samples prepared in solvent. The analyte/IS ratios were used for Pro-Hyp, Hyp-Gly, Gly-Pro-Hyp, Pro-Gly, and Gly-Pro quantification.

### 2.4 Statistics

The data were processed and analyzed with GraphPad Prism 5.04 (Boston, Massachusetts, United States). From the individual concentration time curves, the pharmacokinetic parameters were calculated: incremental area under the curve (iAUC_0–360 min_), delta C_max_ and T_max_. The iAUC values were calculated according to the linear trapezoidal method. The pharmacokinetic data were compared between the different sources as well as between the two different molecular weights CH of bovine origin. Data are presented descriptively with mean and standard deviation (SD). Paired t-tests were performed. Significant differences are indicated with (* *p* < 0.05; ** *p* < 0.01, ***p < 0.001). The summary concentration-time curves with mean values for each of the sampling points are derived from the baseline corrected individual curves.

## 3 Results

### 3.1 Study collective

Six healthy non-smoking volunteers (3 women and 3 men) participated in the study. Characteristics of the study group are summarized in Table 1. None of the volunteers reported to take any chronic regular medication. All volunteers completed the study as planned. All study products were well tolerated. No adverse events were reported during the study days. None of the recorded vital signs and blood routine parameters were judged as clinically significant, what confirms the healthy status of the participants.

**Table 1:** Characteristics of volunteers.

	Mean (SD)
Age [years]	25.2 (7.5)
Body mass index [kg/m^2^]	21.7 (3.0)
Systolic blood pressure [mmHg]	119.8 (14.8)
Diastolic blood pressure [mmHg]	74.3 (8.9)
Total cholesterol [mg/dL]	172.3 (29.5)

### 3.2 Characterization of the study products

The content of free and total (sum of free and peptide-bound) Hyp and of the selected di- and tripeptides present in the study products is summarized in Table 2. The content of free Hyp in each CH product was very low (approximately one thousandth) in comparison to the respective content of total Hyp, which was determined after total hydrolysis of all peptide bonds during sample preparation. The selected peptides were present in free form in a range comparable to free Hyp with the dipeptide Gly-Pro being the most abundant followed by Hyp-Gly which is confirmed for all collagen sources.

**Table 2:** Content of free Hyp, total Hyp as well as selected peptides in the study products.

AAs/Peptides	Fish CH LMW	Porcine CH LMW	Bovine CH LMW	Bovine CH HMW
	[μg/g]	[μg/g]	[μg/g]	[μg/g]
Free Hyp	26.8	33.8	21.0	13.0
Total Hyp	104,000	120,000	108,000	103,000
Pro-Hyp	< 0.8	6.11	3.91	2.08
Hyp-Gly	14.5	24.7	9.97	15.8
Gly-Pro-Hyp	5.97	8.94	2.70	1.71
Pro-Gly	< 8	9.04	9.68	9.61
Gly-Pro	42.5	53.6	40.9	79.5

AAs, amino acids; Hyp, hydroxyproline; Pro-Hyp, proline-hydroxyproline; Hyp-Gly, hydroxyproline-glycine; Gly-Pro-Hyp, glycine-proline-hydroxyproline; Pro-Gly, proline-glycine; Gly-Pro, glycine-proline; CH, collagen hydrolysate; LMW, low mean molecular weight (~2,000 Da); HMW, high mean molecular weight (~5,000 Da).

### 3.3 Oral intake of collagen hydrolysates results in elevated systemic Hyp levels—in both free and peptide-bound form

**Figure 1:**
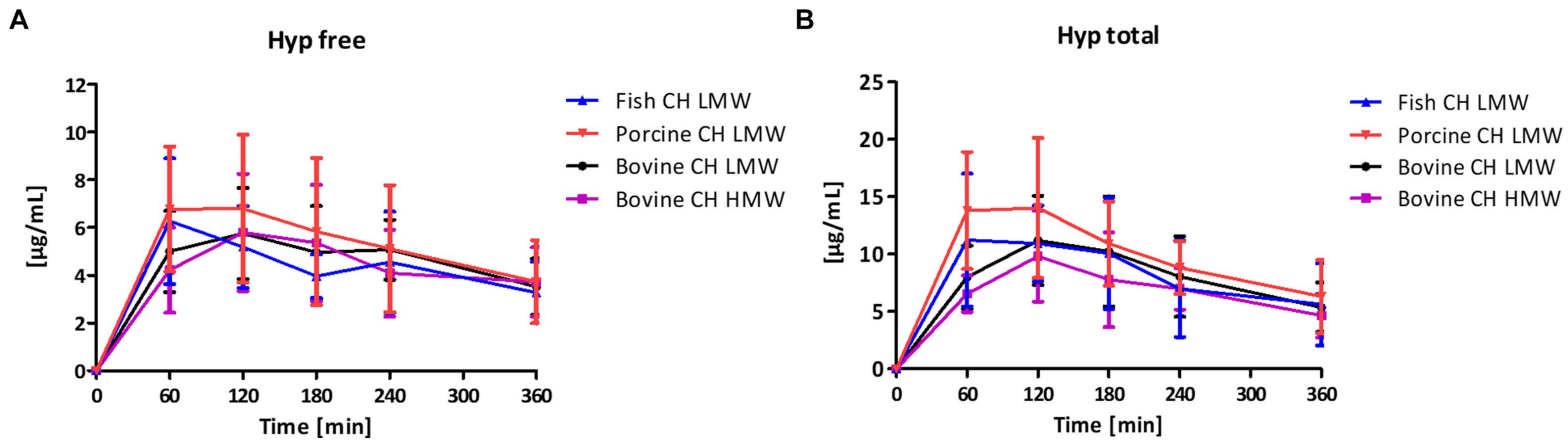
Baseline corrected concentration-time curves of the free hypdroxyproline (Hyp) response **(A)** and total Hyp response **(B)** [μg/mL] after intake of study products (mean ± SD); *n* = 6.

**Figure 2:**
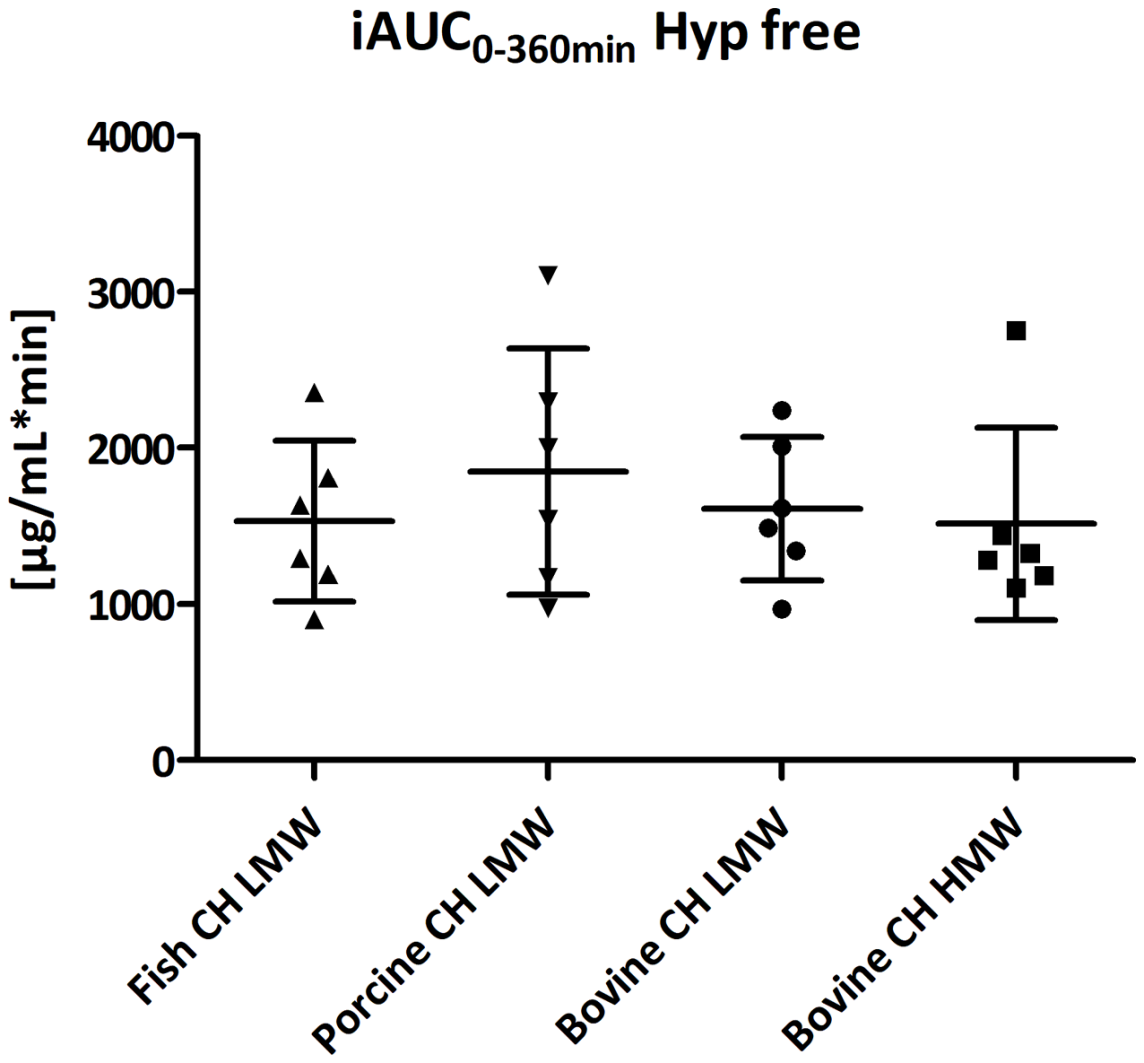
Distribution of iAUC_0–360 min_ min of free Hyp [μg/mL*min] after intake of study products (Scatter diagram with mean ± SD), *n* = 6.

Baseline concentrations of free Hyp in blood were comparable with 1.05, 0.86, 1.18, and 1.14 μg/mL prior the intake of fish CH, porcine CH, bovine CH LMW and bovine CH HMW, respectively. By intake of 10 g CH, free Hyp concentrations in plasma (ΔC_max_) were greatly increased (Figure 1A) with an average factor of 7.2 for fish, 9.9 for porcine and 6.2 for both bovine CH LMW and HMW. With respect to the bioavailability of free Hyp there were no significant differences between the iAUC of the investigated products (Figure 2).

**Figure 3:**
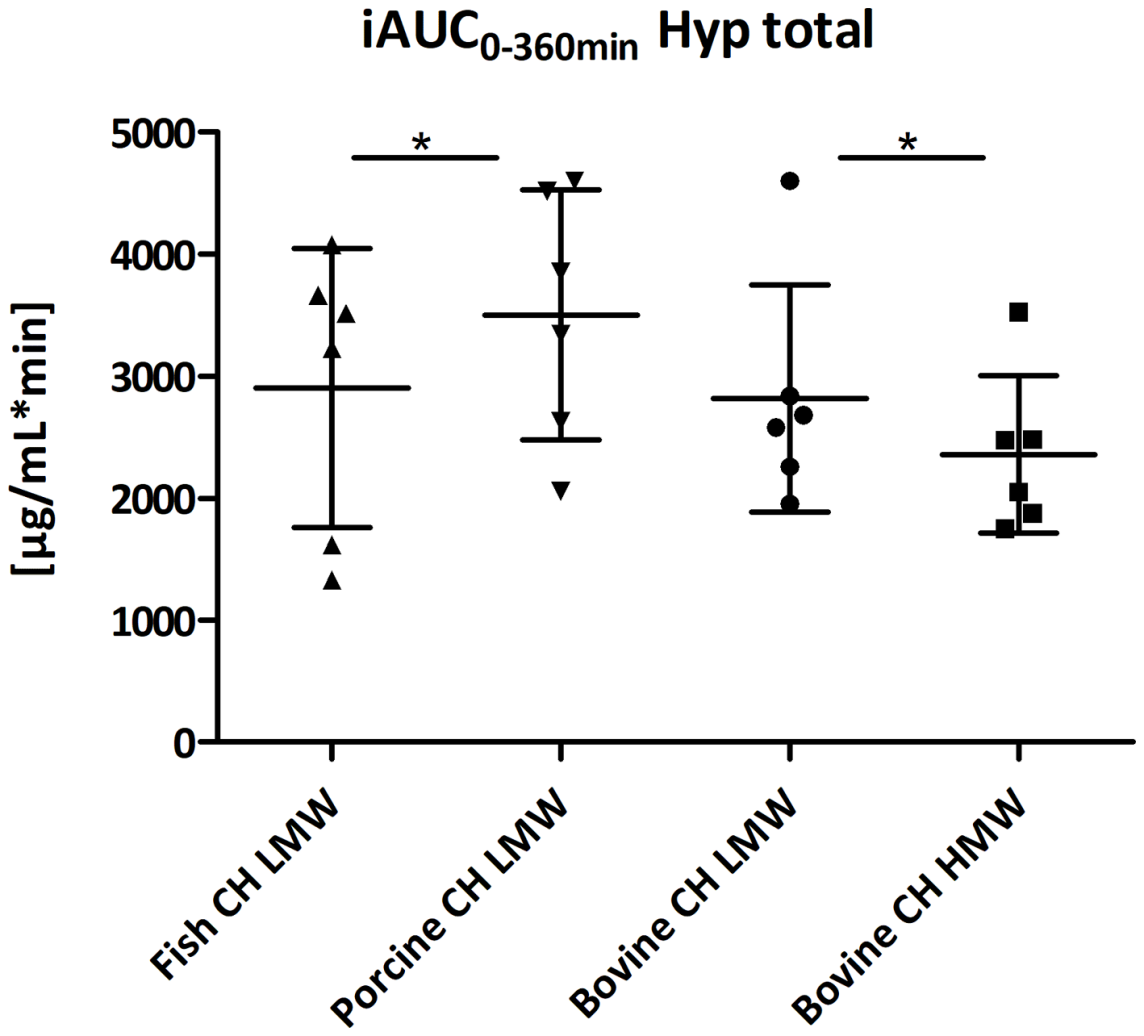
Distribution of iAUC_0–360 min_ min of total Hyp [μg/mL*min] after intake of study products (Scatter diagram with mean ± SD); *p < 0.05; *n* = 6.

In addition, total Hyp content in blood samples was determined after total hydrolysis (Figure 1B). Significantly higher concentrations of total Hyp were determined comparing the iAUC of free and total Hyp (iAUC total Hyp compared to iAUC free Hyp: 47% higher iAUC total Hyp for both fish and porcine CH, 43% for bovine CH LMW and 36% for bovine CH HMW, respectively). In contrast to iAUC for free Hyp, significant differences were observed between the iAUCs for total Hyp: porcine CH was significantly higher in comparison to fish CH (1.2-fold, *p* = 0.0292) and bovine CH LMW was significantly higher in comparison to the bovine CH HMW (1.2-fold, *p* = 0.0262) (Figure 3).

### 3.4 Ingestion of collagen hydrolysates gives rise to elevated levels of bioactive peptides

**Figure 4:**
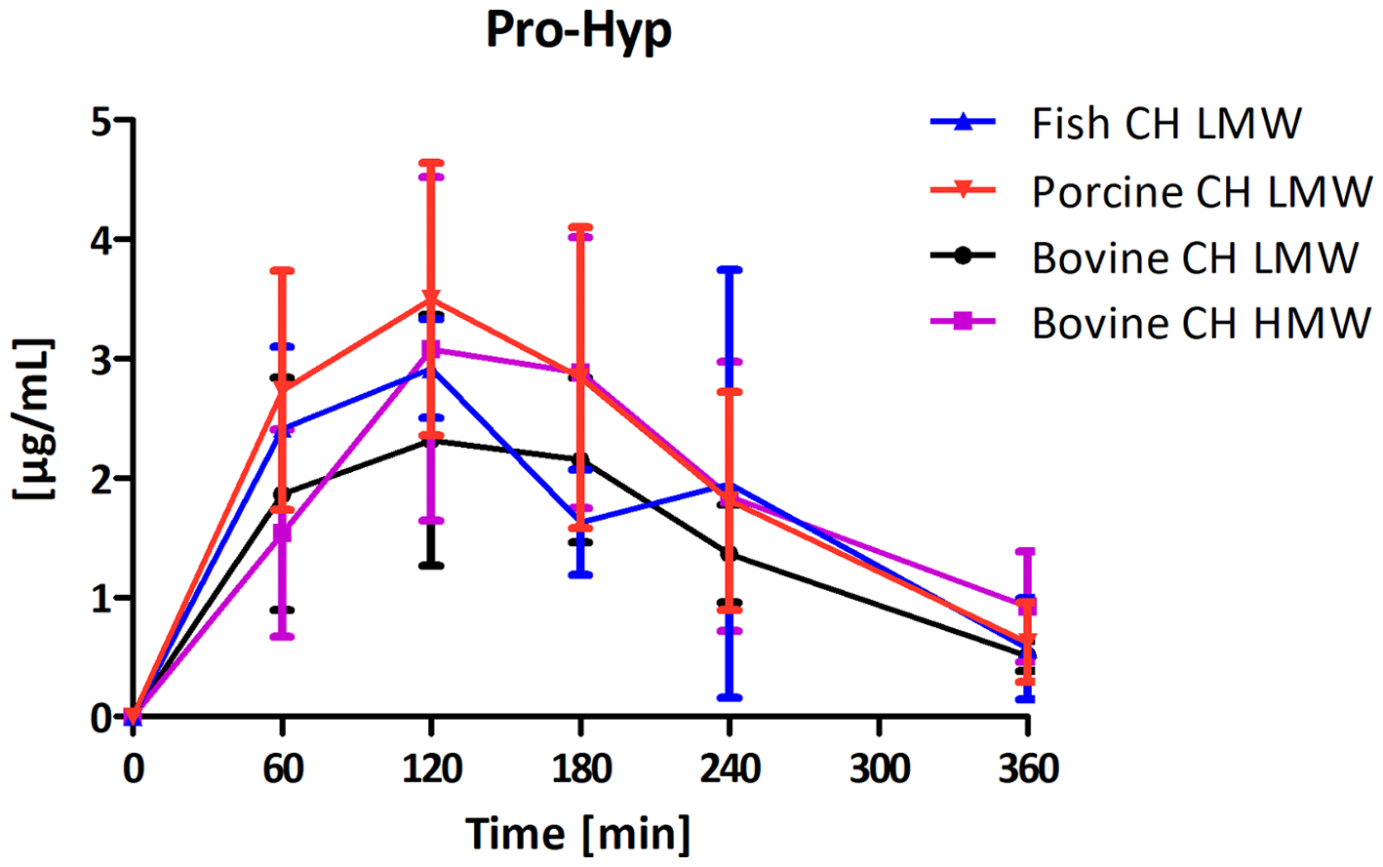
Baseline corrected concentration-time curves of the proline-hydroxyproline (Pro-Hyp) response [μg/mL] after intake of study products (mean ± SD); *n* = 6.

**Figure 5:**
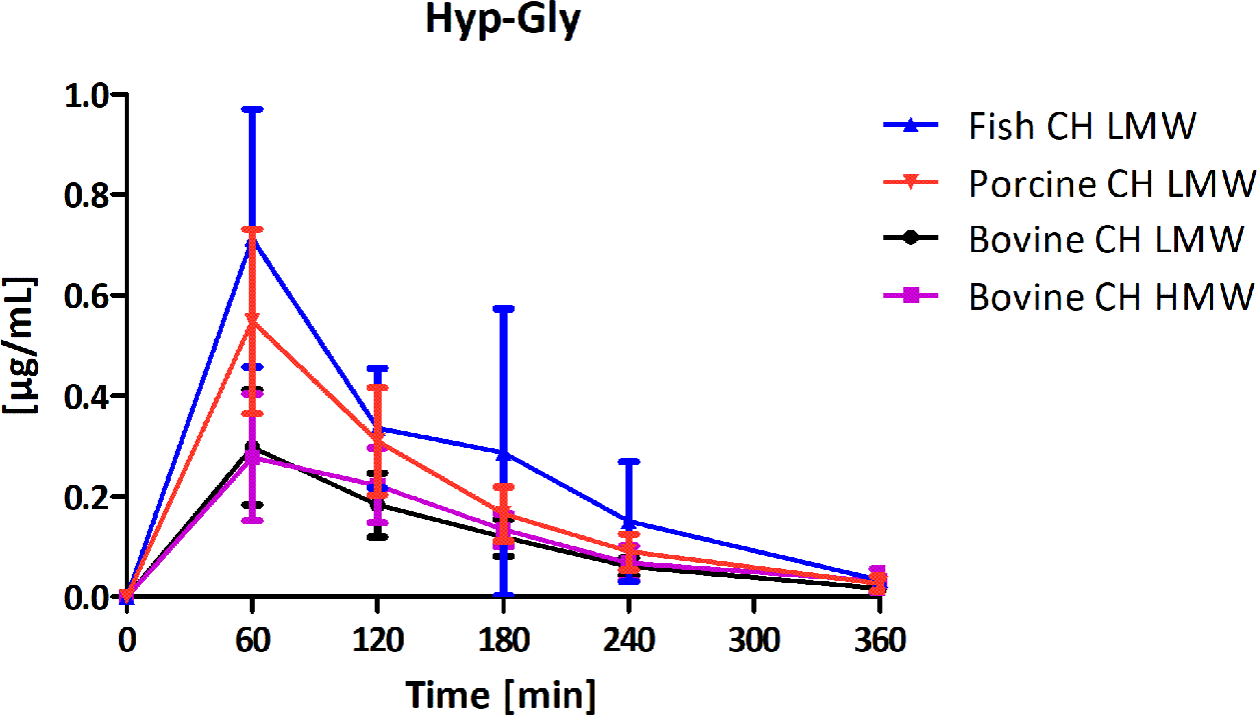
Baseline corrected concentration-time curves of the hydroxyproline-glycine (Hyp-Gly) response [μg/mL] after intake of study products (mean ± SD); *n* = 6.

**Figure 6:**
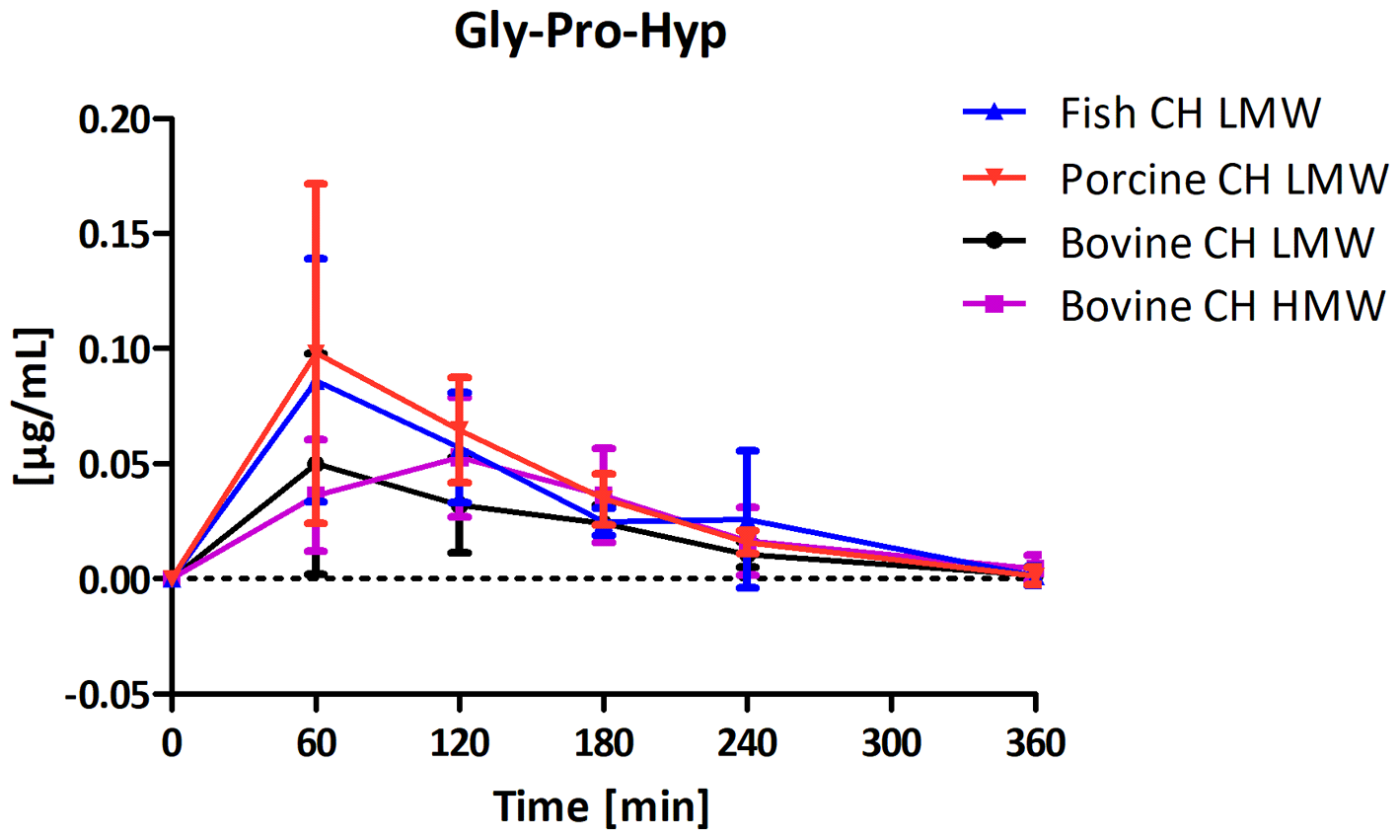
Baseline corrected concentration-time curves of the glycine-proline-hydroxyproline (Gly-Pro-Hyp) response [μg/mL] after intake of study products (mean ± SD); *n* = 6; *n* = 6.

**Figure 7:**
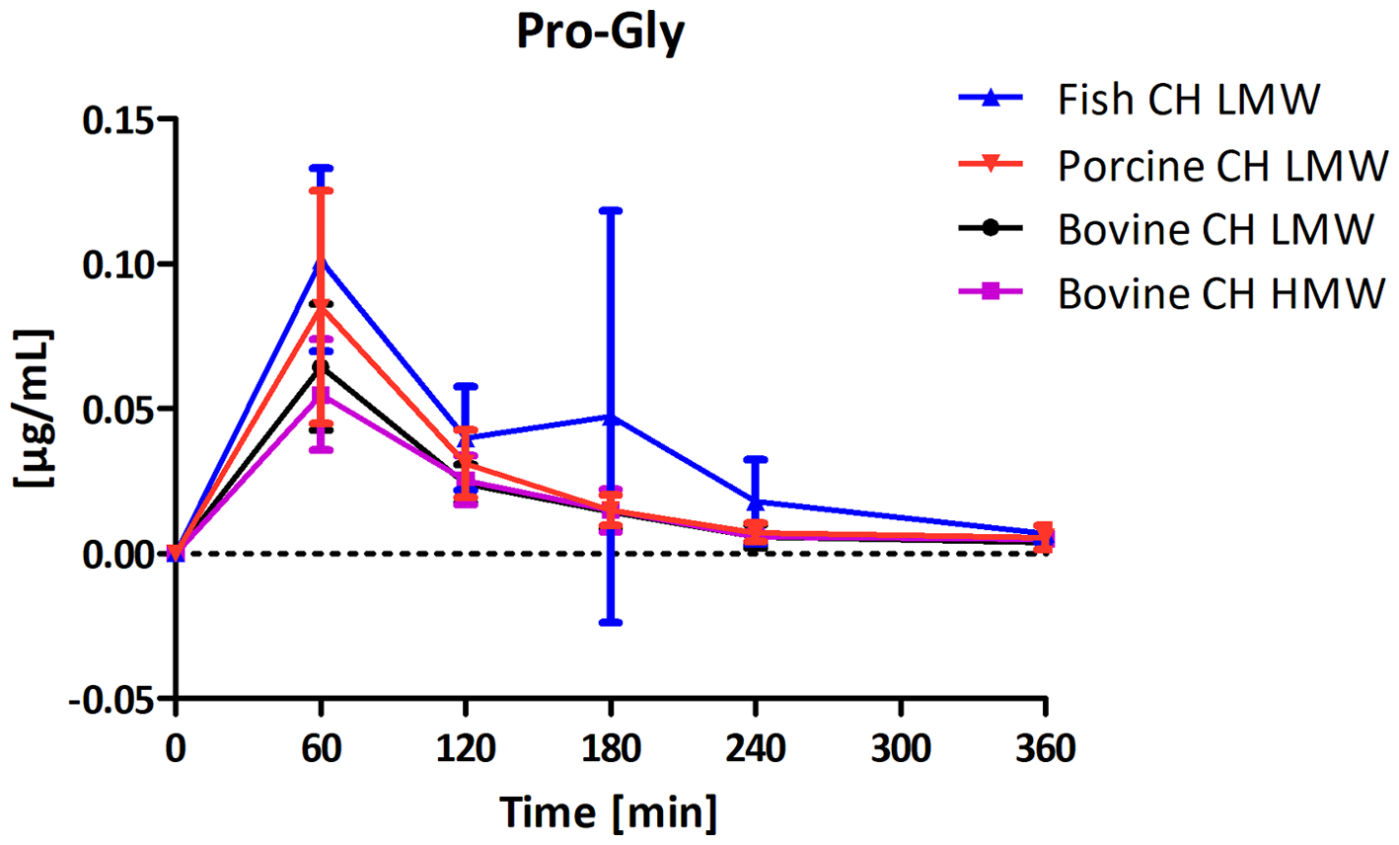
Baseline corrected concentration-time curves of the proline-glycine (Pro-Gly) response [μg/mL] after intake of study products (mean ± SD); *n* = 6.

**Figure 8:**
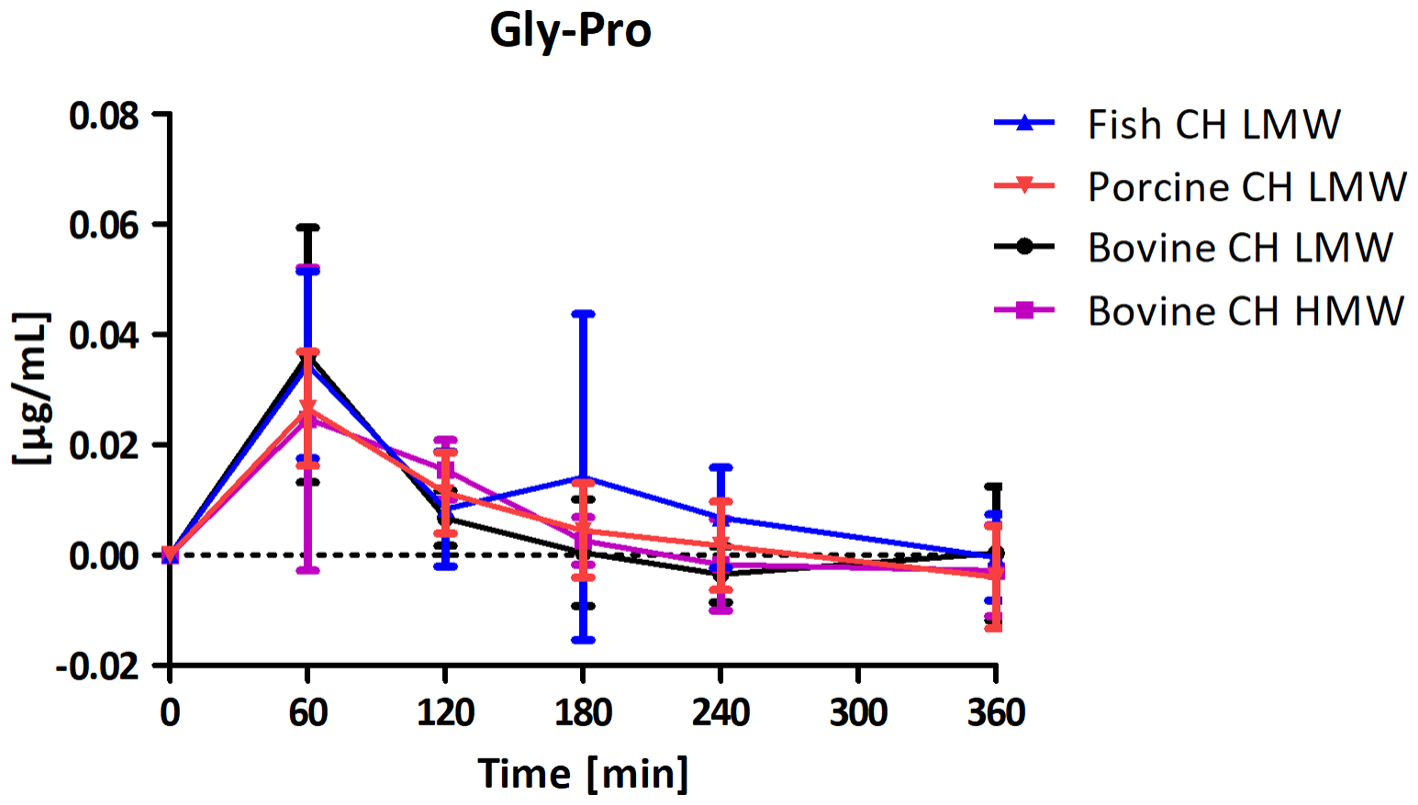
Baseline corrected concentration-time curves of the glycine-proline (Gly-Pro) response [μg/mL] after intake of study products (mean ± SD); *n* = 6.

**Figure 9:**
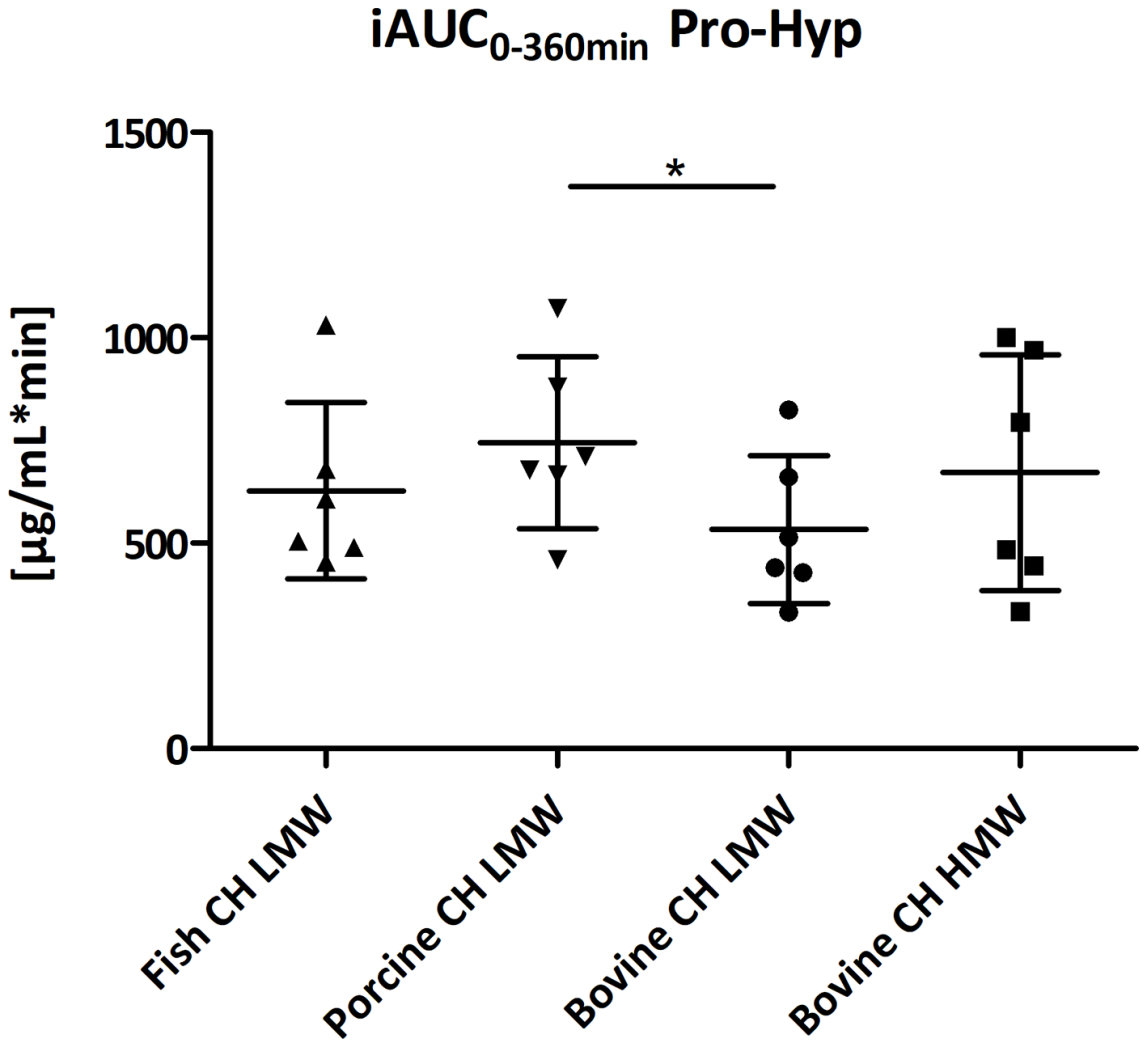
Distribution of iAUC_0–360 min_ of proline-hydroxyproline (Pro-Hyp) [μg/mL*min] after intake of study products (Scatter diagram with mean ± SD); *p < 0.05; *n* = 6.

Among the peptides selected for analysis, Pro-Hyp was the most abundant peptide present in the circulation after intake of CH (Figure 4) compared to Hyp-Gly (Figure 5), Gly-Pro-Hyp (Figure 6), Pro-Gly (Figure 7), and Gly-Pro (Figure 8). Baseline concentrations of Pro-Hyp in blood were comparable at around 0.2 μg/mL. Significant differences in formation and uptake of Pro-Hyp between the different CH sources were only seen between porcine CH LMW vs. bovine CH LMW (1.4-fold; *p* = 0.0159, see Figure 9). No differences were seen for the uptake characteristics of Pro-Hyp between the bovine CH LMW and HMW.

**Figure 10:**
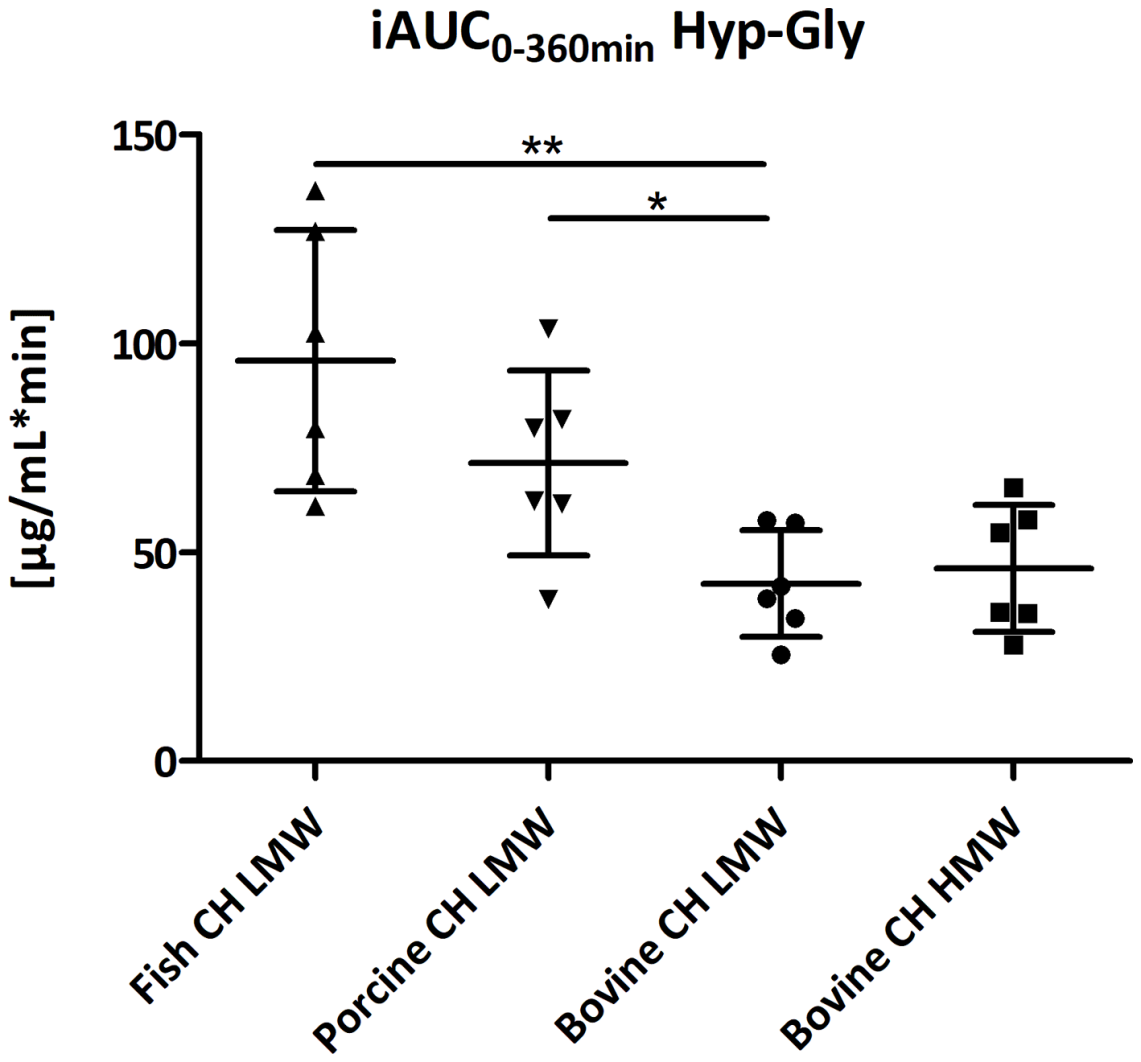
Distribution of iAUC_0–360 min_ min of hydroxyproline-glycine (Hyp-Gly) [μg/mL*min] after intake of study products (Scatter diagram with mean ± SD); *p < 0.05, **p < 0.01; *n* = 6.

Formation and absorption of Hyp-Gly after intake of different CH sources was highest for the fish CH (Figure 5) with statistically significant differences between fish CH and bovine CH LMW (2.3-fold; *p* = 0.0050) and porcine CH in comparison to bovine CH LMW (1.7-fold; *p* = 0.0178) (Figure 10). No differences were seen for the uptake characteristics of Hyp-Gly between the bovine CH LMW and HMW.

**Figure 11:**
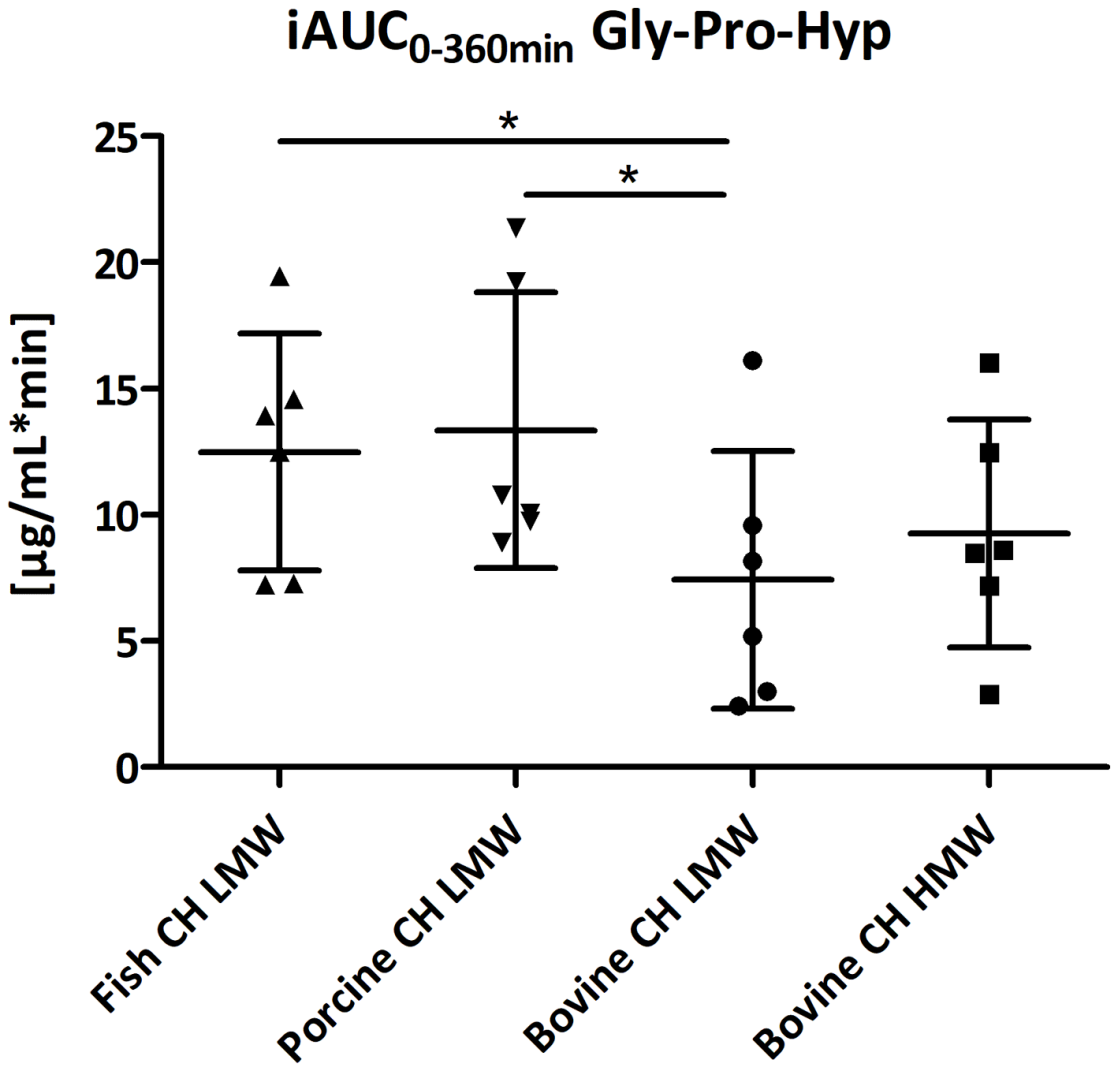
Distribution of iAUC_0–360 min_ of glycine-proline-hydroxyproline (Gly-Pro-Hyp) [μg/mL*min] after intake of study products (Scatter diagram with mean ± SD); *p < 0.05; *n* = 6.

**Figure 12:**
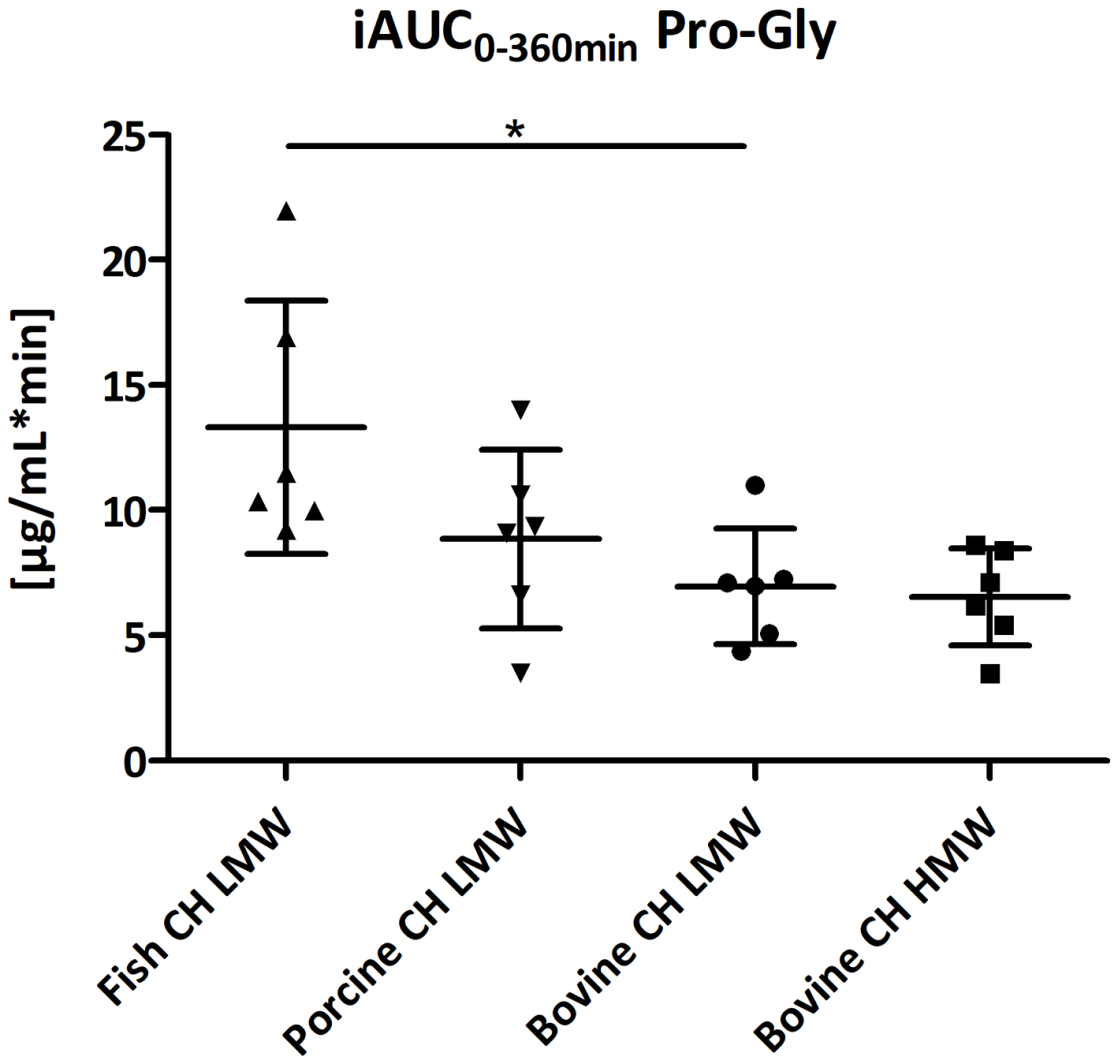
Distribution of iAUC_0–360 min_ min of proline-glycine (Pro-Gly) [μg/mL*min] after intake of study products (Scatter diagram with mean ± SD); *p < 0.05; *n* = 6.

Gly-Pro-Hyp and Pro-Gly were formed and absorbed into the blood at a much lower concentration in comparison to Pro-Hyp and Hyp-Gly (Figures 4–7). However, for both statistically significant differences were observed between fish CH in comparison to bovine CH LMW [1.7-fold; *p* = 0.0307 (Gly-Pro-Hyp); 1.9-fold; *p* = 0.0373 (Pro-Gly)] (Figures 11, 12). In addition, the uptake of Gly-Pro-Hyp from porcine CH LMW was significantly higher in comparison to the bovine CH LMW (1.8-fold; *p* = 0.0309, see Figure 11). No differences were seen for the uptake characteristics of Gly-Pro-Hyp and Pro-Gly between the bovine CH LMW and HMW.

**Figure 13:**
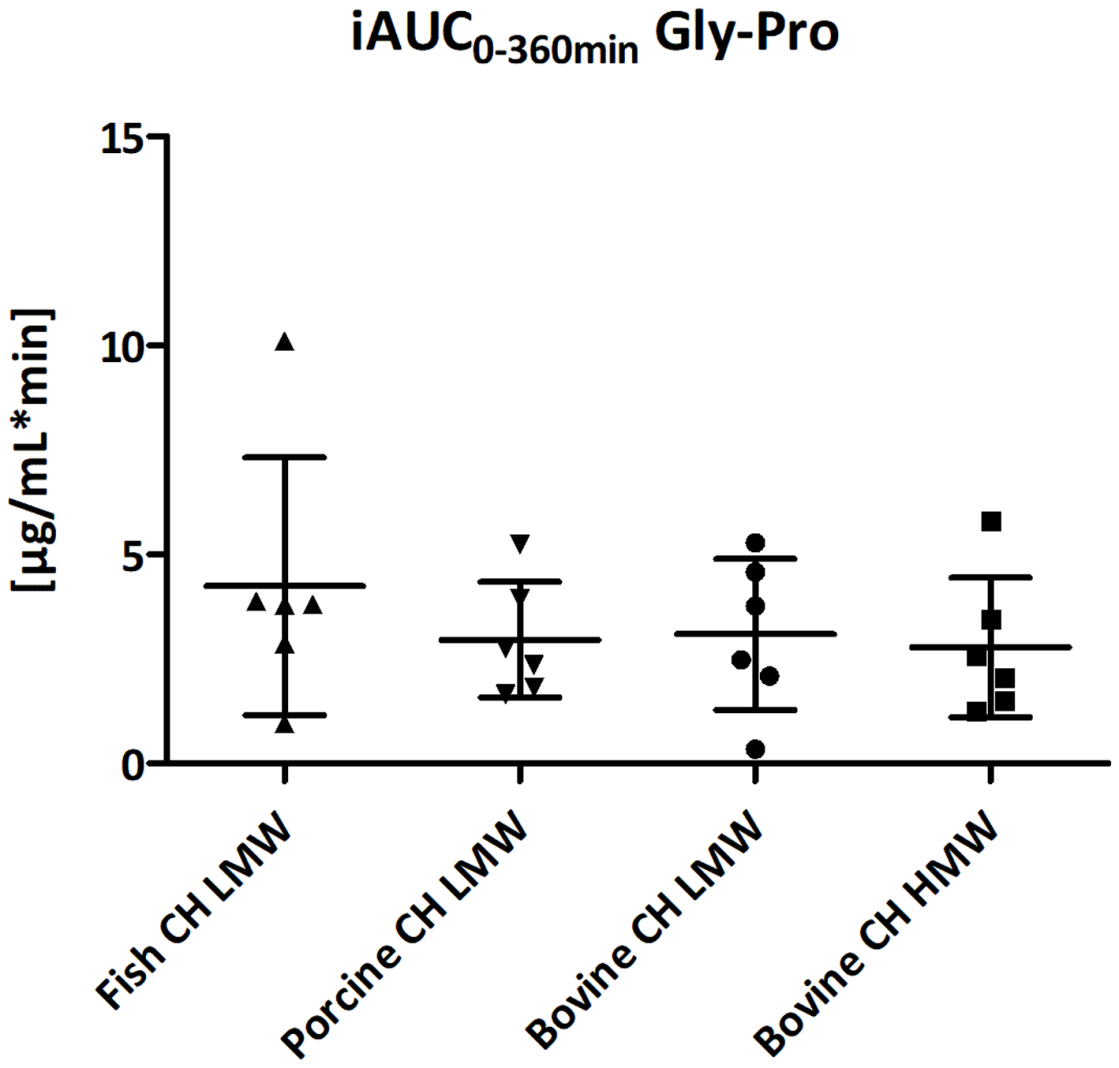
Distribution of iAUC_0–360 min_ of glycine-proline (Gly-Pro) [μg/mL*min] after intake of study products (Scatter diagram with mean ± SD); *n* = 6.

The dipeptide Gly-Pro was found to be less abundant in the blood samples compared to the other dipeptides (Figures 4–8). However, it had the highest concentration among all dipeptides in the study products prior to ingestion. No differences in formation and uptake of Gly-Pro were observed comparing the different raw material sources (fish, porcine and bovine). Furthermore, bioavailability of Gly-Pro from bovine CH LMW and bovine CH HMW were comparable (Figure 13).

## 4 Discussion

Bioavailability refers to the fraction of a nutrient or bioactive compound that can be absorbed into the bloodstream from food upon consumption and is available for use by the body. Factors such as food processing, food composition, genetic differences and individual variations in gastrointestinal digestion and absorption can affect nutrient bioavailability (38, 39). For CH products ongoing discussions focus on how elements of the manufacturing process, raw material origin and degree of hydrolysis (which determines the mean molecular weight) influence differences in bioavailability between CH products and whether these differences translate into varying bioactivity.

Due to its scarce presence in animal protein, Hyp is considered as the signature AAs for collagen and is used as a marker to track the uptake of CH-derived metabolites into the bloodstream (30). In the present study, investigated CH from all sources (bovine, porcine and fish) and mean molecular weights (2,000 Da vs. 5,000 Da) were absorbed, as indicated by a substantial increase in free Hyp, reaching maximum plasma concentrations 6–10 times higher than baseline within 100–130 min after ingestion. Correspondingly, the uptake kinetic patterns measured for total Hyp were very similar for all CH, however with a ΔC_max_ approximately twice as high compared to free Hyp. This finding clearly indicates that a considerable fraction of Hyp remains in peptide-bound form in the circulation—between 36 and 47% depending on the CH investigated –, confirming that CH are digested and absorbed both as free AAs and peptide-bound forms.

Notably, there were minimal variations in the total Hyp content of the CH products before ingestion. After oral intake, statistically significant differences between CH were observed for some, but not all, pharmacokinetic endpoints. Descriptively, porcine CH showed a higher iAUC for total Hyp compared to fish CH. The iAUC of total Hyp, but not free Hyp, or any of the investigated peptides, was significantly lower for the HMW compared to the LMW bovine CH. Remarkably, inter-individual differences in uptake kinetics seemed to be pronounced as indicated by the spread of the individual data points for the iAUC of some parameters (e.g., total Hyp, Figure 3, or Hyp-Gly, Figure 10), which may partially contribute to the observed differences.

The cross-over design of this study controlled for confounding factors such as genetic constitution, gut motility, digestive enzyme activity, gut mucosa composition, and gut microbiome composition and functionality to the maximum extent possible. However, the pilot scale sample size limits the generalization of the observed differences. Further studies with a larger sample size are warranted to confirm the findings.

The observed levels of free and total Hyp align with information currently reported in the scientific literature (36, 40). In a study with five volunteers, Ohara et al. compared the uptake of three sources of CH from fish scale, fish skin, or porcine skin with the same mean molecular weight and reported a contribution of Hyp-containing peptides to total Hyp uptake of about 30% (36). In the current study, this fraction was estimated to be even higher between 36 and 47% corresponding to a substantial part of all absorbed Hyp. *In situ* intestinal perfusion analysis suggested that CH was absorbed predominantly as peptides, showing that the peptides could pass through the liver and reach the systemic circulation (41). Similar to findings in this study, Ohara et al. reported a slightly higher AUC for porcine skin CH compared to fish skin CH (1.1-fold total Hyp), which was significant for free Hyp (1.5-fold). Additionally, fish scale CH resulted in a different uptake profile with significantly higher AUC for free and total Hyp compared to fish skin CH (36). Despite the same animal source, it is known that fish scale has a different collagen composition than fish skin potentially explaining the differences in absorption (42).

Targeted analysis of the selected peptides in plasma revealed that Pro-Hyp was the most abundant peptide detected in this study after intake of the different CH sources. Intake of 10 g yielded high plasma concentrations (e.g., porcine CH ΔC_max_ 3.8 μg/mL) despite Pro-Hyp being the peptide with the lowest average abundance in the CH products prior to ingestion. These results clearly indicate that this peptide is formed and released during digestion and absorption in the gastrointestinal tract by cleavage from larger oligopeptides. In contrast, the peptide Gly-Pro had the highest abundance in the CH products prior to ingestion but resulted in a ΔC_max_ 100 times lower than that of Pro-Hyp.

Thus, the initial peptide content of the CH products is not a direct indicator for the expected blood concentrations of a given peptide. Literature supports this, showing substantial increases of peptides in plasma after single-dose ingestion as reported by Kleinnijenhuis et al. (5). In addition, CH products with a different mean molecular weight, bovine HMW (~5,000 Da) and LMW (~2,000 Da), led to highly comparable peptide and AAs concentrations in blood. These observations indicate that di- and tripeptides are further released during digestion and absorption.

This is also supported by Iwasaki et al., who showed that a CH (89% components <3,000 Da) significantly enhanced plasma concentrations of Pro-Hyp and Hyp-Gly compared to gelatin, which has a high molecular weight (100,000 Da). The absorption of free Hyp following gelatin ingestion (94.4 ± 16.4 nmol/mL) was significantly lower compared to CH (169.1 ± 32.5 nmol/mL) (35), indicating that the dipeptides were cleaved from the parent collagen protein during gastrointestinal passage, but that a very high initial molecular weight of gelatin and the complex physicochemical properties of gelling collagen reduces the peptide’s bioavailability.

Current literature shows that Pro-Hyp appears at very high concentrations after oral intake of CH (porcine skin, chicken feet and cartilage origin) reaching maximum concentrations after 60–120 min, consistent with our findings (4). Others confirmed Pro-Hyp as the most abundant peptide following ingestion of CH from porcine skin, fish skin and scale (36, 43). Shigemura et al. reported extensive variation between volunteers in the ratio of the dipeptides Pro-Hyp and Hyp-Gly, but only fish scale derived CH was investigated (43). In the present study, the ratio of Pro-Hyp to Hyp-Gly averaged 12.6 for the bovine CH LMW, 14.6 for the bovine CH HMW but only 6.6 for the fish CH and 10.5 for the porcine CH.

For the other investigated peptides, Hyp-Gly had much lower plasma concentrations than Pro-Hyp for all products (ΔC_max_ range: 0.29–0.76 μg/mL). For this dipeptide, there was a clear difference between products with fish CH presenting a significantly higher C_max_ compared to bovine CH LMW (*p* = 0.0112), and porcine CH (*p* = 0.0412). The other investigated peptides Gly-Pro-Hyp, Pro-Gly, and Gly-Pro were less abundant in plasma.

Despite differences in individual peptides between some of the CH investigated in the present study, the results indicate that the absorption of fish, porcine and bovine CH is highly comparable. Some peptides have been linked to specific bioactivities, but due to the complexity of the product (oligopeptide mix) and metabolites in the blood it is not possible to establish a simple cause-effect relationship to explain the biological benefits of CH. Relating these findings to biological activity was beyond the scope of this investigation, but general biological effects have been shown for diverse CH products, suggesting they can be used interchangeably for certain health applications. Benefits for skin health have been reported for CH of fish, porcine and bovine sources (19, 21, 44). The efficacy of CH from fish skin, porcine skin, or bovine hide was confirmed by Wauquier et al. regarding bone cell activity using CH-metabolite containing human serum *in vitro* (15). Furthermore, in a clinical study with osteoarthritis patients, both CH from porcine and bovine origin confirmed the ability to improve osteoarthritis symptoms with no reported differences between sources (12).

The present study compared the uptake profiles of different CH products in response to a single dose application. The complexity of CH-derived metabolite profiles in the blood might increase during long-term CH supplementation. According to Shigemura et al., daily ingestion of CH for a longer period, i.e., 4 weeks, changed the profile of Hyp-containing peptides in human blood (45). The compositional rate of Hyp-Gly tended to increase, while Pro-Hyp tended to decrease after daily ingestion. It was speculated that long-term CH ingestion might change exo- or endo-type protease activity in the digestive tract, potentially promoting beneficial effects. Further investigation is warranted to determine how the bioavailability of total and peptide-bound Hyp predicts the uptake kinetics and bioavailability of specific bioactive peptides, and how this is linked to CH’s bioactivity.

In the present study, selected CH-derived peptides described to be among the most abundant, were investigated for their bioavailability. Larger scale studies are needed to improve the understanding of how inter-individual physiological variability and differences in CH product characteristics may impact the bioavailability of bioactive components relevant to CH bioactivity. Further investigations are also warranted to extend the knowledge of the mechanisms underlying CH’s health benefits.

## 5 Conclusion

Substantial knowledge gaps exist on how different CH products compare regarding their digestion, bioavailability, and bioactivity, with bovine-derived CH being underrepresented in current research in particular.

In the present study the uptake of free Hyp was not affected by the source or molecular weight of the CH. However, the bioavailability of the peptides Hyp-Gly, Pro-Hyp, Gly-Pro-Hyp, and Pro-Gly from CH of different sources showed significant differences, indicating an impact of the animal species and potential inter-individual variation. On the other hand, the molecular weight of the CH did not affect the bioavailability of the selected peptides.

It is noteworthy that a growing body of literature reports health benefits for different CH products, regardless of their source or molecular weight. This suggests that certain products may be used interchangeably. However, further research is needed to understand the complex matrix connecting (1) the CH product origin (animal source, molecular weight, enzymatic hydrolysis process), (2) the metabolite patterns that appear in the circulation after CH ingestion, to (3) the bioactivity in the target tissues and the potential overall health benefit. For this purpose, defining the threshold concentrations of individual circulating metabolites and their potential interaction needed to trigger distinct physiological reactions in different target tissues are a crucial step. This would allow a better comparison of different CH products and help to predict their efficacy. In addition, investigating the impact of the food matrix on the CH efficacy is of importance, considering that CH are ingredients in functional foods and supplements.

## Data availability statement

The raw data supporting the conclusions of this article will be made available by the authors, without undue reservation.

## Ethics statement

The studies involving humans were approved by Landesärztekammer Baden-Württemberg (F-2019-075). The studies were conducted in accordance with the local legislation and institutional requirements. The participants provided their written informed consent to participate in this study.

## Author contributions

NV: Writing – original draft, Conceptualization, Validation, Writing – review & editing. CS: Investigation, Software, Validation, Writing – review & editing, Conceptualization, Formal analysis, Project administration, Visualization, Writing – original draft. YM: Investigation, Software, Validation, Writing – review & editing. BS: Writing – review & editing, Data curation. SV: Writing – original draft. FH: Methodology, Writing – review & editing. AK: Methodology, Writing – review & editing. CIFS: Writing – original draft. JP: Writing – original draft, Funding acquisition, Resources, Supervision.

## References

[1] Larder CE Iskandar MM Kubow S. Collagen hydrolysates: a source of bioactive peptides derived from food sources for the treatment of osteoarthritis. Medicines. (2023) 10:50. doi: 10.3390/medicines10090050 37755240 PMC10538231

[2] Xu X Wang D Li J Zeng X Zhang Z Zhu J. Collagen hydrolysates from deer tendon: preparation assisted with different ultrasound pretreatment times and promotion in MC3T3-E1 cell proliferation and antioxidant activities. Process Biochem. (2023) 133:228–40. doi: 10.1016/j.procbio.2023.09.010

[3] Zhang SY Zhao YQ Wang YM Yang XR Chi CF Wang B. Gelatins and antioxidant peptides from skipjack tuna (*Katsuwonus pelamis*) skins: purification, characterization, and cytoprotection on ultraviolet-a injured human skin fibroblasts. Food Biosci. (2022) 50:102138–8. doi: 10.1016/j.fbio.2022.102138

[4] Iwai K Hasegawa T Taguchi Y Morimatsu F Sato K Nakamura Y. Identification of food-derived collagen peptides in human blood after oral ingestion of gelatin hydrolysates. J Agric Food Chem. (2005) 53:6531–6. doi: 10.1021/jf050206p, PMID: 16076145

[5] Kleinnijenhuis AJ van Holthoon FL Maathuis AJ Vanhoecke B Prawitt J Wauquier F. Non-targeted and targeted analysis of collagen hydrolysates during the course of digestion and absorption. Anal Bioanal Chem. (2020) 412:973–82. doi: 10.1007/s00216-019-02323-x, PMID: 31872275 PMC7005076

[6] Spanier B Rohm F. Proton coupled oligopeptide transporter 1 (PepT1) function, regulation, and influence on the intestinal homeostasis. Compr Physiol. (2018) 8:843–69. doi: 10.1002/cphy.c170038, PMID: 29687907

[7] Akbarian M Khani A Eghbalpour S Uversky VN. Bioactive peptides: synthesis, sources, applications, and proposed mechanisms of action. Int J Mol Sci. (2022) 23:1445. doi: 10.3390/ijms23031445, PMID: 35163367 PMC8836030

[8] Edgar S Hopley B Genovese L Sibilla S Laight D Shute J. Effects of collagen-derived bioactive peptides and natural antioxidant compounds on proliferation and matrix protein synthesis by cultured normal human dermal fibroblasts. Sci Rep. (2018) 8:10474. doi: 10.1038/s41598-018-28492-w, PMID: 29992983 PMC6041269

[9] Nuñez SM Guzmán F Valencia P Almonacid S Cárdenas C. Collagen as a source of bioactive peptides: a bioinformatics approach. Electron J Biotechnol. (2020) 48:101–8. doi: 10.1016/j.ejbt.2020.09.009

[10] Yazaki M Ito Y Yamada M Goulas S Teramoto S Nakaya M-A. Oral ingestion of collagen hydrolysate leads to the transportation of highly concentrated Gly-Pro-Hyp and its hydrolyzed form of pro-Hyp into the bloodstream and skin. J Agric Food Chem. (2017) 65:2315–22. doi: 10.1021/acs.jafc.6b05679, PMID: 28244315

[11] Dar Q-A Schott EM Catheline SE Maynard RD Liu Z Kamal F. Daily oral consumption of hydrolyzed type 1 collagen is chondroprotective and anti-inflammatory in murine posttraumatic osteoarthritis. PLoS One. (2017) 12:e0174705. doi: 10.1371/journal.pone.0174705, PMID: 28384173 PMC5383229

[12] Kumar S Sugihara F Suzuki K Inoue N Venkateswarathirukumara S. A double-blind, placebo-controlled, randomised, clinical study on the effectiveness of collagen peptide on osteoarthritis. J Sci Food Agric. (2015) 95:702–7. doi: 10.1002/jsfa.6752, PMID: 24852756

[13] Zdzieblik D Oesser S Gollhofer A Konig D. Improvement of activity-related knee joint discomfort following supplementation of specific collagen peptides. Appl Physiol Nutr Metab. (2017) 42:588–95. doi: 10.1139/apnm-2016-0390, PMID: 28177710

[14] Guillerminet F Fabien-Soulé V Even PC Tomé D Benhamou C-L Roux C. Hydrolyzed collagen improves bone status and prevents bone loss in ovariectomized C3H/HeN mice. Osteoporos Int. (2012) 23:1909–19. doi: 10.1007/s00198-011-1788-6, PMID: 21927918

[15] Wauquier F Daneault A Granel H Prawitt J Fabien Soulé V Berger J. Human enriched serum following hydrolysed collagen absorption modulates bone cell activity: from bedside to bench and vice versa. Nutrients. (2019) 11:1249. doi: 10.3390/nu11061249, PMID: 31159319 PMC6627680

[16] Zhang L Zhang S Song H Li B. Effect of collagen hydrolysates from silver carp skin (*hypophthalmichthys molitrix*) on osteoporosis in chronologically aged mice: increasing bone remodeling. Nutrients. (2018) 10:1434. doi: 10.3390/nu10101434, PMID: 30287779 PMC6212965

[17] Centner C Jerger S Mallard A Herrmann A Varfolomeeva E Gollhofer S. Supplementation of specific collagen peptides following high-load resistance exercise upregulates gene expression in pathways involved in skeletal muscle signal transduction. Front Physiol. (2022) 13:838004. doi: 10.3389/fphys.2022.838004, PMID: 35480041 PMC9037237

[18] Clifford T Ventress M Allerton DM Stansfield S Tang JCY Fraser WD. The effects of collagen peptides on muscle damage, inflammation and bone turnover following exercise: a randomized, controlled trial. Amino Acids. (2019) 51:691–704. doi: 10.1007/s00726-019-02706-5, PMID: 30783776

[19] Asserin J Lati E Shioya T Prawitt J. The effect of oral collagen peptide supplementation on skin moisture and the dermal collagen network: evidence from an ex vivo model and randomized, placebo-controlled clinical trials. J Cosmet Dermatol. (2015) 14:291–301. doi: 10.1111/jocd.12174, PMID: 26362110

[20] Inoue N Sugihara F Wang X. Ingestion of bioactive collagen hydrolysates enhance facial skin moisture and elasticity and reduce facial ageing signs in a randomised double-blind placebo-controlled clinical study. J Sci Food Agric. (2016) 96:4077–81. doi: 10.1002/jsfa.7606, PMID: 26840887

[21] Maia Campos PMBG Oliveira de Melo M Mendes Fossa Shirata M Gabarra Leite M. Oral intake of bioactive collagen peptides in the improvement of skin and hair: clinical studies by instrumental measurements. Biomed Biopharm Res. (2022) 19:379–96. doi: 10.19277/bbr.19.2.297

[22] Larder CE Iskandar MM Kubow S. Assessment of bioavailability after in vitro digestion and first pass metabolism of bioactive peptides from collagen hydrolysates. Curr Issues Mol Biol. (2021) 43:1592–605. doi: 10.3390/cimb43030113, PMID: 34698092 PMC8928955

[23] Sato K Jimi S Kusubata M. Generation of bioactive prolyl-hydroxyproline (Pro-Hyp) by oral administration of collagen hydrolysate and degradation of endogenous collagen. Int J Food Sci Technol. (2019) 54:1976–80. doi: 10.1111/ijfs.14145

[24] Knight CG Morton LF Onley DJ Peachey AR Ichinohe T Okuma M. Collagen-platelet interaction: Gly-pro-Hyp is uniquely specific for platelet Gp VI and mediates platelet activation by collagen. Cardiovasc Res. (1999) 41:450–7. doi: 10.1016/s0008-6363(98)00306-x, PMID: 10341844

[25] Ichimura T Yamanaka A Otsuka T Yamashita E Maruyama S. Antihypertensive effect of enzymatic hydrolysate of collagen and Gly-pro in spontaneously hypertensive rats. Biosci Biotechnol Biochem. (2009) 73:2317–9. doi: 10.1271/bbb.90197, PMID: 19809172

[26] Takemori K Yamamoto E Ito H Kometani T. Prophylactic effects of elastin peptide derived from the bulbus arteriosus of fish on vascular dysfunction in spontaneously hypertensive rats. Life Sci. (2015) 120:48–53. doi: 10.1016/j.lfs.2014.10.011 25445217

[27] Martynova KV Andreeva LA Klimova PA Kirillova IG Shevchenko VP Nagaev II. Structural-functional study of glycine-and-proline-containing peptides (glyprolines) as potential neuroprotectors. Bioorg Khim. (2009) 35:165–71. doi: 10.1134/S1068162009020022, PMID: 19537167

[28] Gorres KL Raines RT. Prolyl 4-hydroxylase. Crit Rev Biochem Mol Biol. (2010) 45:106–24. doi: 10.3109/10409231003627991, PMID: 20199358 PMC2841224

[29] Shoulders MD Raines RT. Collagen structure and stability. Annu Rev Biochem. (2009) 78:929–58. doi: 10.1146/annurev.biochem.77.032207.120833, PMID: 19344236 PMC2846778

[30] Cissell DD Link JM Hu JC Athanasiou KA. A modified hydroxyproline assay based on hydrochloric acid in ehrlich's solution accurately measures tissue collagen content. Tissue Eng Part C Methods. (2017) 23:243–50. doi: 10.1089/ten.tec.2017.0018, PMID: 28406755 PMC5397204

[31] Wu G Bazer FW Burghardt RC Johnson GA Kim SW Knabe DA. Proline and hydroxyproline metabolism: implications for animal and human nutrition. Amino Acids. (2011) 40:1053–63. doi: 10.1007/s00726-010-0715-z, PMID: 20697752 PMC3773366

[32] Misiura M Miltyk W. Proline-containing peptides-new insight and implications: a review. Biofactors. (2019) 45:857–66. doi: 10.1002/biof.1554 31430415

[33] Taga Y Kusubata M Ogawa-Goto K Hattori S Funato N. Collagen-derived X-Hyp-Gly-type tripeptides promote differentiation of MC3T3-E1 pre-osteoblasts. J Funct Foods. (2018) 46:456–62. doi: 10.1016/j.jff.2018.05.017

[34] Taga Y Iwasaki Y Tometsuka C Funato N Shigemura Y Kusubata M. Identification of a highly stable bioactive 3-hydroxyproline-containing tripeptide in human blood after collagen hydrolysate ingestion. NPJ Sci Food. (2022) 6:29. doi: 10.1038/s41538-022-00144-4, PMID: 35662250 PMC9166765

[35] Iwasaki Y Nakatogawa M Shimizu A Sato Y Shigemura Y. Comparison of gelatin and low-molecular weight gelatin hydrolysate ingestion on hydroxyproline (Hyp), pro-Hyp and Hyp-Gly concentrations in human blood. Food Chem. (2022) 369:130869. doi: 10.1016/j.foodchem.2021.130869, PMID: 34461513

[36] Ohara H Matsumoto H Ito K Iwai K Sato K. Comparison of quantity and structures of hydroxyproline-containing peptides in human blood after oral ingestion of gelatin hydrolysates from different sources. J Agric Food Chem. (2007) 55:1532–5. doi: 10.1021/jf062834s, PMID: 17253720

[37] Kleinnijenhuis AJ Vergauwen B van Holthoon FL Hekman M. Reliable and generic liquid chromatography/mass spectrometry quantification of short peptides using a stable-isotope-labeled labeling agent. Rapid Commun Mass Spectrom. (2020) 34:e8934. doi: 10.1002/rcm.8934, PMID: 32885531

[38] Parada J Aguilera JM. Food microstructure affects the bioavailability of several nutrients. J Food Sci. (2007) 72:R21–32. doi: 10.1111/j.1750-3841.2007.00274.x, PMID: 17995848

[39] Sun X Acquah C Aluko RE Udenigwe CC. Considering food matrix and gastrointestinal effects in enhancing bioactive peptide absorption and bioavailability. J Funct Foods. (2020) 64:103680. doi: 10.1016/j.jff.2019.103680

[40] Wu G. Important roles of dietary taurine, creatine, carnosine, anserine and 4-hydroxyproline in human nutrition and health. Amino Acids. (2020) 52:329–60. doi: 10.1007/s00726-020-02823-6, PMID: 32072297 PMC7088015

[41] Osawa Y Mizushige T Jinno S Sugihara F Inoue N Tanaka H. Absorption and metabolism of orally administered collagen hydrolysates evaluated by the vascularly perfused rat intestine and liver in situ. Biomed Res. (2018) 39:1–11. doi: 10.2220/biomedres.39.1, PMID: 29467346

[42] Jafari H Lista A Siekapen MM Ghaffari-Bohlouli P Nie L Alimoradi H. Fish collagen: extraction, characterization, and applications for biomaterials engineering. Polymers. (2020) 12:2230. doi: 10.3390/polym12102230, PMID: 32998331 PMC7601392

[43] Shigemura Y Akaba S Kawashima E Park EY Nakamura Y Sato K. Identification of a novel food-derived collagen peptide, hydroxyprolyl-glycine, in human peripheral blood by pre-column derivatisation with phenyl isothiocyanate. Food Chem. (2011) 129:1019–24. doi: 10.1016/j.foodchem.2011.05.066, PMID: 25212331

[44] Ohara H Ichikawa S Matsumoto H Akiyama M Fujimoto N Kobayashi T. Collagen-derived dipeptide, proline-hydroxyproline, stimulates cell proliferation and hyaluronic acid synthesis in cultured human dermal fibroblasts. J Dermatol. (2010) 37:330–8. doi: 10.1111/j.1346-8138.2010.00827.x, PMID: 20507402

[45] Shigemura Y Suzuki A Kurokawa M Sato Y Sato K. Changes in composition and content of food-derived peptide in human blood after daily ingestion of collagen hydrolysate for 4 weeks. J Sci Food Agric. (2018) 98:1944–50. doi: 10.1002/jsfa.8677, PMID: 28914450

